# Medical History from the Earliest Times

**Published:** 1893-07-08

**Authors:** E. T. Withington


### Medical History from the Earliest Times.

By E. T. Withington, M.A., M.B. Oxon.

### LXI.—THE ORGANICISTS.

We have now considered two of the three forms of medical theory which replaced the chemical and mechanical systems of the seventeenth century, and may here briefly repeat the definition given in a previous chapter. The Animists, Vitalists, and Organicists agreed in maintaining that vital activity cannot be explained by the laws of physics and chemistry, but while the two former held that life is due to some spiritual entity residing in the body, and acting upon it like an engineer on his engine, the Organicists believed that it results from the intimate structure of the body itself. The objection that mere structure without a force behind it must be purely passive may be met in two ways: (1) By replying that the active agency is to be sought not in some unknown metaphysical abstraction, but in the perpetual stimulus of external nature, or in modern phraseology, that life is a continuous series of reflex actions; and (2) by pointing out that matter and force are inseparably connected, and that even so-called dead substance possesses unknown capabilities of action which may be manifested only under special conditions. The history of organicism shows the gradual development of these two theories till they are at last clearly formulated in the systems of Brown and Reil respectively, and we may trace their common origin in the doctrine of "irritability," first taught by Francis Glisson (1597—1677), Regius Professor of Medicine at Cambridge.

Glisson, whose name is best known from his description of rickets, and his researches on the anatomy of the liver, considered that the most striking characteristic of living tissue is the way in which it responds to stimuli. This he called "irritability," distinguished various kinds and degrees of it, and attributed it not only to the solids, but even to some of the fluids of the body; life, in short, is irritability. With regard to animals, Glisson seems to have believed that irritability is an inherent property of their tissues, but he hesitated probably from theological scruples to apply the same doctrine to man, in whose case he proposed another theory, more nearly allied to that of the Animists and Vitalists. The irritability of the human body, he declared, is due to some semi-material substance resembling it in shape, separable from it at death, and the special seat of the immortal soul. It is thus very like the orthodox idea of an angel, and Glisson points out that our conceptions of those mysterious beings are closely allied to our ideas of life, "for who can imagine a dead angel!"

But Glisson's theory was so vague and contradictory, and so mixed up with philosophic subtleties, that it attracted little attention till the doctrine of irritability was revived in modified form by the greatest physician of the eighteenth century, Albert von Haller. Haller made valuable contributions to almost every department of medicine as well as to other sciences, but we can only consider here his two papers "On the Sentient and Irritable Parts of Animals," read before the Royal Society of Göttingen, April 22nd and May 6th, 1752, the most important physiological productions since Harvey's "Anatomical Exercise." Relying upon numerous vivisections, "undertaken with great reluctance in the hope of benefiting the human race," Haller says that he has arrived at a new classification of animal structures into irritable, sentient, and neutral. He points out that the two former properties are distinct from one another, and that irritability, the chief objective manifestation of life, is not only independent of sensibility, but survives for a time the death of the animal, and is shown in parts separated from the rest of the body. This was an attack both on the old theory of "animal spirits" and the modern doctrines of the Animists and Vitalists, and opponents sprang up on all sides, the ablest of whom was the semi-Animist, Whytt, of Edinburgh. But even Whytt confesses that the experiments of Haller force us to believe either that the head, body, and other parts of a frog may retain their "anima" or sentient principle when separated from each other, or "that the active powers of animals are merely properties of the kind of matter of which they are made." He prefers the former alternative, as being more in accordance with theology, but a dissected soul, or chopped-up sentient principle is even harder to imagine than a dead angel, and Whytt is driven at last to the usual resort of the mystic, "What are we that we should presume to limit by our narrow and inadequate capacities the powers of incorporeal natures?" Haller, however, stuck manfully to his facts, and his final triumph not only settled the question as to whether the healing art was to be based on metaphysical theory or scientific observation and experiment, but through the labours of Bichat, which were its direct result, contributed largely to that revolution in medicine which marks the beginning of the present century.

Practical physicians were naturally more attracted by the doctrine of the organicists, than by vague theories which converted diseases into unknown derangements of some equally unknown "principle," and the "neuropathology" of Cullen and Bordeu may be looked upon as an attempt to combine the teaching of Haller with the older views of the "Solidists," Baglivi and Hoffmann. Cullen thought that the distinction of irritability and sensibility might be removed by considering muscle as the direct continuation of nerve. Life could then be defined as a property of the nervous system, or nervous energy. The causes of disease act by increasing or diminishing the excitability of this nerve force and thereby produce changes in the body, especially in the form of spasm or atony. The therapeutic indications are to remove the exciting cause, to treat the lesions produced, and to maintain the nervous energy, or, in more general language, "to obviate the tendency to death."

We must pass over Cullen's many other services to medicine to speak for a moment of one who was first his pupil and afterwards his opponent. It seemed to John Brown, M.D., St. Andrews, absurd to trouble oneself either with the observations of Cullen or the experiments of Haller when the great word "Excitability," if properly manipulated, will not only explain all the phenomena of life and disease, but also afford a simple and universal rule of treatment; and he proceeded to propound a medical system of such peculiarity and interest that we must discuss it in a separate chapter. Stated in the fewest possible words, the theory was this: The animal body possesses a property called excitability, which, when acted on by stimuli, gives rise to excitement, or, in other words, life. Moderate excitement is health; too much or too little excitement is disease, which is to be treated by regulating the stimuli.

Meanwhile the progress of science, especially the discovery of oxygen and increased knowledge of electricity, threw a new light upon the capacities of so-called dead matter. The chemists and physicists began to return to the attack with improved weapons and united forces, and they found an unexpected ally in John Christian Reil, a pupil of Haller, whose name is immortalised in the human brain. Reil not only held that the active powers of the body are properties of its substance, but further asserted that the distinction of a special set of vital forces, and another inferior set of chemical and physical forces, is untenable. Volta had shown that by arranging metallic plates in a certain order, the marvellous force called electricity was developed. Why should not the infinitely more complex arrangement of matter in the human body give rise to yet more wonderful activities? Indeed, may not the body, or at any rate the nervous system, consist of an infinite number of microscopic Voltaic piles or galvanic batteries? And Reil, carried away by the closeness of the analogy, finally declared that Life is Electricity. His followers went still further. The development of positive and negative electricity at the opposite ends of a Voltaic pile or battery, is expressed by the term "polarity." Here was a still finer word than Brown's excitability, for it might be applied not only to the body, but to the whole universe. Irritability is positive, sensibility negative; health is positive, disease negative; man is positive, woman negative; mind is positive, matter negative, &c., and Polarity, with a big P., in some way helps to explain it all.

